# Formin 1-Isoform IV Deficient Cells Exhibit Defects in Cell Spreading and Focal Adhesion Formation

**DOI:** 10.1371/journal.pone.0002497

**Published:** 2008-06-18

**Authors:** Markus Dettenhofer, Fen Zhou, Philip Leder

**Affiliations:** Department of Genetics, Harvard Medical School, Boston, Massachusetts, United States of America; Dresden University of Technology, Germany

## Abstract

**Background:**

Regulation of the cytoskeleton is a central feature of cell migration. The formin family of proteins controls the rate of actin nucleation at its barbed end. Thus, formins are predicted to contribute to several important cell processes such as locomotion, membrane ruffling, vesicle endocytosis, and stress fiber formation and disassociation.

**Methodology/Principal Findings:**

In this study we investigated the functional role of Formin1-isoform4 (Fmn1-IV) by using genetically null primary cells that displayed augmented protrusive behaviour during wound healing and delayed cell spreading. Cells deficient of Fmn1-IV also showed reduced efficiency of focal adhesion formation. Additionally, we generated an enhanced green fluorescence protein (EGFP)-fused Fmn1-IV knock-in mouse to monitor the endogenous subcellular localization of Fmn1-IV. Its localization was found within the cytoplasm and along microtubules, yet it was largely excluded from adherens junctions.

**Conclusions/Significance:**

It was determined that Fmn1-IV, as an actin nucleator, contributes to protrusion of the cell's leading edge and focal adhesion formation, thus contributing to cell motility.

## Introduction

Eukaryotic cells utilize actin for a variety of specialized locomotor functions. As a cell senses its environment, filopodia and lamellipodia are projected forward and extracellular cues are monitored by cell surface receptors. Actin additionally participates in the trafficking of vesicles that transport membrane-associated cargo to and away from the cell surface. To date, three classes of actin nucleators control the rate of actin filament extension, the Arp2/3 complex, Spire, and the formins. The formin family of proteins consists of approximately 25 members, with the forming homology 2 (FH2) domain defining family membership. The formins are expressed ubiquitously in eukaryotic cells [1]–[3]. The FH2 of the yeast Formin, Bni1 [4], [5] was originally shown to be sufficient for the nucleation of actin filaments at its barbed end, and subsequently FH2 domains derived from other formins have been shown to function similarly [6]–[8]. Crystal structure analyses have further suggested that FH2 domains function as dimers [9] and as nucleators of actin onto existing actin filaments [10], [11]. Moreover, the FH2 domain acts by interfering with capping protein's gaining access to the growing actin filament, which leads to a model of formins acting as leaky cappers [12]. In addition to the FH2 domain, the majority of formins possess a forming homology 1 (FH1) domain that is highly proline-rich and functions in recruiting profilin to the emerging actin filament [13]–[15].

Although the mechanism by which formins nucleate actin is becoming clearer, an understanding of their spatial and temporal regulation is just emerging. Several formins act in response to the Rho family of GTPases [16], [17]. Dia1 functions through a dynamic self-folding mechanism that acts as an auto-inhibitory domain [18] to regulate stress fiber formation [19], [20] and stabilize microtubules [21]. Moreover, recent knockdown studies convincingly demonstrated the role of Dia1 in stress fiber elongation [22]. While much has been learned about the Diaphanous-related formins (DRF), very little is known about the regulation of the founding member of the formin family, Formin1. Formin 1 consists of several splice variants that are differentially expressed in mammalian tissues [23]–[26]. The most widely expressed variant is Fmn1-IV, which we have previously knocked-out in mice and found to result in weakly penetrant kidney aplasia [27]. Since they also possess an actin nucleation domain, functional regulation of the 6 different mRNA isoforms (Ia, Ib, II, III, IV, and V) of Fmn1 will in part be defined by the cell type in which they are expressed, as well as by the different peptide sequences they encode to determine their respective subcellular localizations. Formin1-isoform1, which is highly expressed in the trigeminal and dorsal root ganglia [26], [27] localizes to microtubules through a peptide encoded by exon 2 [28]. By examining primary cells derived from EGFP fused to Fmn1-IV knock-in mice we determined that the localization of Fmn1-IV was within the cytoplasm, and not significantly concentrated at adherens junctions. More specifically, Fmn1-IV also localizes along microtubules. Primary cells derived from Fmn1-IV knock-out mice demonstrated altered processive behavior at the cell periphery coupled with a delay in cell spreading and altered focal adhesion formation.

## Results

### Fmn1-IV localizes to the cytoplasm and along microtubules

**Figure 1:**
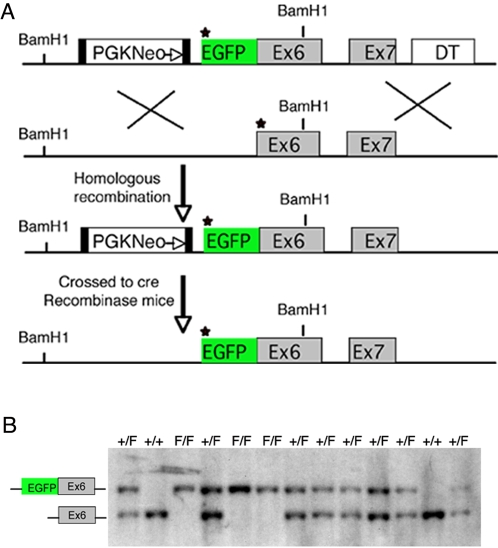
Generation of the EGFP-Fmn1-IV fusion allele. (A) Diagram of the strategy for generating an in-frame insertion of EGFP into the Fmn1-IV locus, which produces an EGFP-tagged Fmn1-IV driven from the endogenous promoter in mice. The star designates the start of translation. The black filled boxes flanking the PGK Neo cassette represent LoxP sites. (B) Representative PCR genotyping of tail DNA from mice possessing the EGFP-Fmn1-IV allele are shown. The letter F indicates the fusion allele, while + indicates wt allele.

In this study, we examined the subcellular localization and functional role of Fmn1-IV, which has a broad pattern of tissue expression, including the limb bud, kidney, and gonad, and is distinguished from the other Formin1 isoforms by the inclusion of exon 6 within its coding region [25], [26]. It has been shown that the FH1-FH2 domain of Fmn1 nucleates actin filaments *in vitro* [29]. Since the detection of endogenous Fmn1-IV has been difficult with the existing reagents, we generated an EGFP-Fmn1-IV fusion knock-in mouse in which the fusion protein is driven from the endogenous promoter (Fig. 1A). A targeting construct was generated by fusing exon 6 of Fmn1 in-frame with EGFP with the start of translation being from the first EGFP methionine. Using embryonic stem cell technology, mice were generated in which Fmn1-IV was replaced by the EGFP-Fmn1-IV fusion protein. Mouse tail DNA was used to screen for the stable insertion of EGFP fused to Fmn1-IV initially by Southern blot analysis and confirmed by PCR (Fig. 1B).

**Figure 2:**
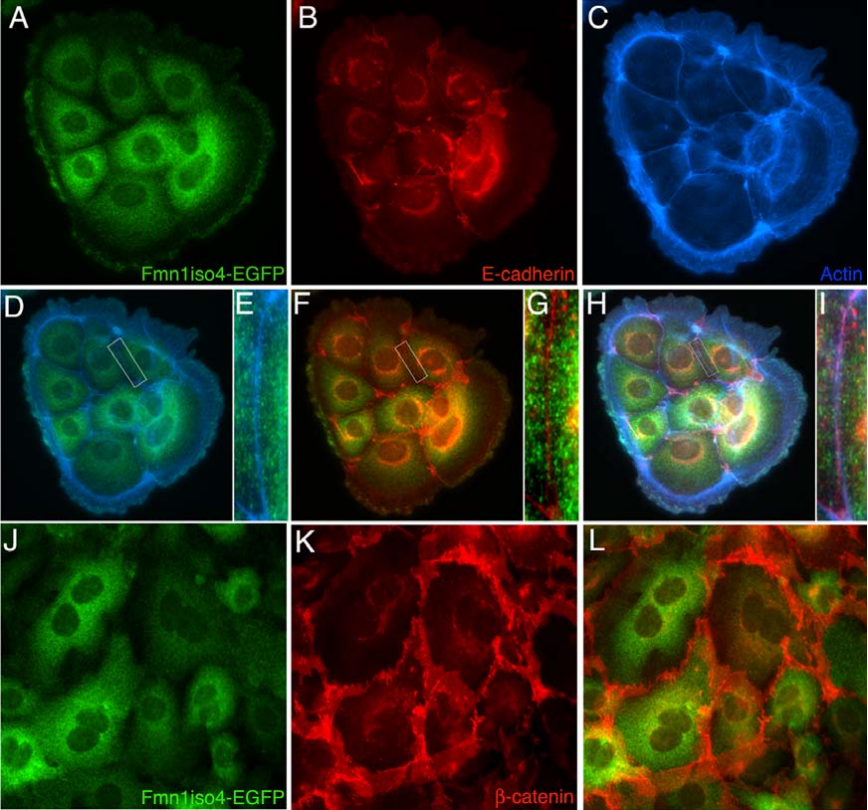
Intracellular localization of Fmn1-IV in primary kidney epithelial cells derived from EGFP-Fmn1-IV knock-in mice (A-L). Immunofluorescence staining of primary epithelial cells with anti-GFP antibody (A), anti-E-cadherin antibody (B), and phalloidin (C). Merged images: (D) anti-GFP and phalloidin (magnified insert, E), (F) anti-GFP and E-cadherin (magnified insert, G), and (H) anti-GFP, phalloidin and E-cadherin (magnified insert, I). Confluent epithelial cells stained with anti-GFP (J), anti-β-catenin (K), and merged image (L). Note that Fmn1-IV is largely absent from adherens junctions, by its lack of co-localization with E-cadherin and β-catenin.

**Figure 3:**
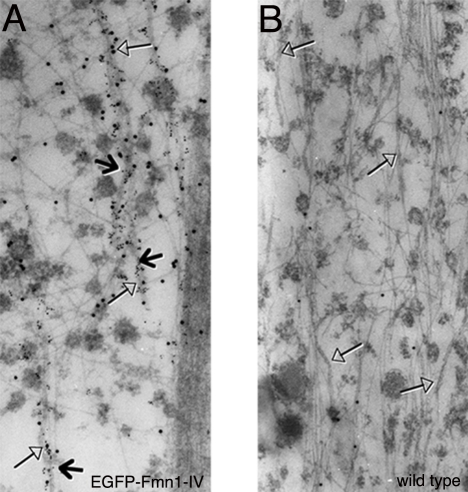
Localization of EGFP-Fmn1-IV by immuno-gold EM in MEFs derived from knock-in mice. (A and B) Tubulin and EGFP are labeled with 6 nm and 15 nm gold particles, respectively. Open head arrows indicate microtubules, and arrows indicate EGFP-Fmn1-IV association along microtubules.

Immunofluorescence analysis of primary epithelial cells derived from kidneys of EGFP-Fmn1-IV mice demonstrated its cytoplasmic localization, which was not significantly enriched in adherens junctions (Fig. 2). Fmn1-IV does not tend to colocalize with the major filamentous actin structures of cells, such as stress fibers or at the leading edge of lamellipodia. To gain a more thorough understanding of the nature of the cytoplasmic localization, immuno-gold electron microscopy was performed on primary MEFs derived from EGFP-Fmn1-IV knock-in and wild type control mice (Fig. 3A and B). These data show an association of EGFP-Fmn1-IV with microtubules.

### Fmn1-IV influences protrusive behavior at the leading edge of cells

**Figure 4:**
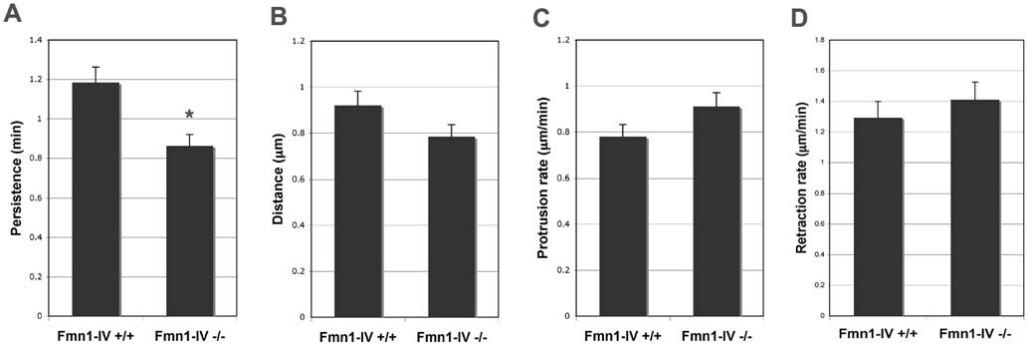
Fmn1-IV −/− MEFs exhibit altered protrusive behavior. Time-lapse microscopic images were taken from the peripheries of representative wild-type and Fmn1-IV −/− MEFs grown on a fibronectin substrate after wound healing. Kymographs of the leading edge of cells were analyzed both for protrusive and retractive behavior. (A) Protrusive persistence (Δx), (B) protrusive distance (Δy), (C) protrusion rate (Δy/Δx), and (D) retraction rate (−Δy/Δx) were determined and the data represent the mean±SEM (error bars). *, P<0.05.

From an overall functional stand-point, it was important to determine what role Fmn1-IV plays in the cell. As an actin nucleator that localizes within the cytoplasm and can associate along microtubules, Fmn1-IV might be involved in lamellipodial regulation. To examine this possibility, we compared the protrusive and retractive behavior from the periphery of wild-type and Fmn1-IV −/− mouse embryo fibroblasts (MEFs) after wound healing on a fibronectin substrate. MEFs were analyzed by kymography of time-lapse videos using ImageJ. Persistence (Δx) and distance (Δy) of protrusion and retraction of active cell lamellipodia were used to derive the protrusion rate (Δy/Δx) and retraction rate (−Δy/Δx). Protrusion rates of Fmn1-IV −/− MEFs trended towards more rapid activity, 0.910±0.104 μm/min compared to wild-type MEFs, 0.780±0.0531 μm/min (Fig. 4C). Rates of retraction similarly trended towards more activity for Fmn1-IV −/− MEFs, 1.41±0.143 μm/min compared to wild-type MEFs, 1.293±0.106 μm/min (Fig. 4D). These trends could be explained by a significant reduction in the persistence of protrusive activity for Fmn1-IV −/− versus wild-type MEFs, 0.861±0.098 min and 1.18±0.081 min, respectively (Fig. 4A). Further the extension of protrusions were 0.920±0.0627 μm for wild-type and 0.784±0.0892 μm for Fmn1-IV −/− MEFs (Fig. 4B). Since the formins are actin nucleators, the observations that MEFs lacking Fmn1-IV have slightly more active protrusion and retraction rates suggests that Fmn1-IV acts to limit cell ruffling. Noting that Fmn1-IV does not significantly accumulate at the periphery of cells, further suggests that its regulatory activity may contribute to other sites of actin nucleation that indirectly play a role in cell protrusive behavior.

### Fmn1-IV enhances cell spreading and focal adhesion formation

**Figure 5:**
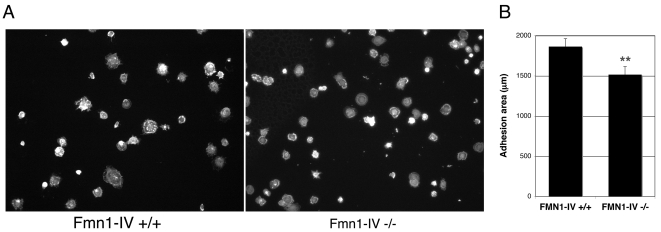
Fmn1-IV −/− MEFs exhibit reduced cell spreading behavior. (A) Wild-type and Fmn1-IV null MEFs were fixed and stained for filamentous actin after 15 min of replating on fibronectin. The total cell area was measured with Openlab. (B) Results shown are the mean±SEM (error bars). **, P<0.001.

**Figure 6:**
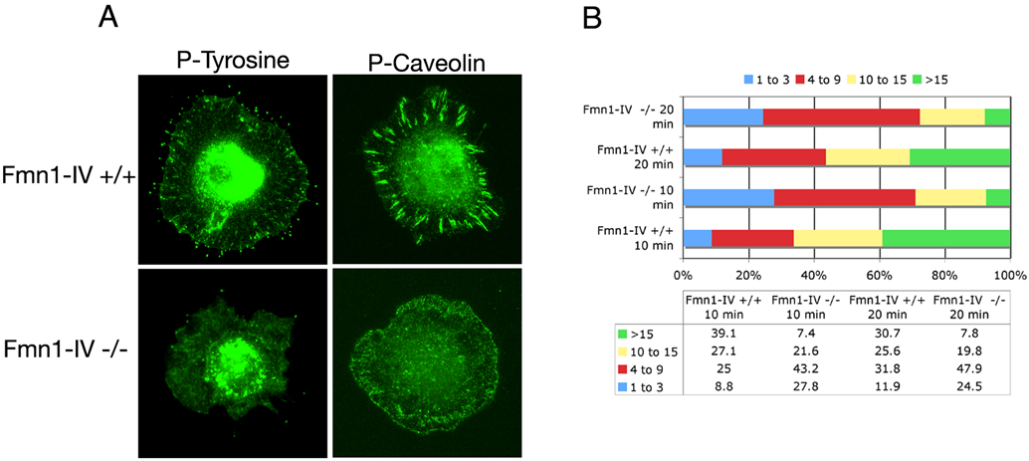
Fmn1-IV −/− MEFs exhibit reduced focal adhesion formation in a cell spreading assay. (A) Wild-type and Fmn1-IV null MEFs were replated on fibronectin substrates for 10 or 20 min before fixation. Focal adhesions were imaged by immunofluorescence after staining with phospho-tyrosine or phospho-caveolin antibodies. (B) The graph represents the percentages of cells (out of 100%) possessing the range of focal adhesions, as indicated. Numbers of cells examined for focal adhesions: Fmn1-IV +/+ 10 min n = 192, Fmn1-IV −/− 10 min n = 162, Fmn1-IV +/+ 20 min n = 176, Fmn1-IV −/− 20 min n = 167.

Cell plating assays on a fibronectin substrate were used to determine the relative cell adhesion efficiencies. After fixation, cells were stained with phalloidin and the area of cell adhesion was measured using Openlab (Fig. 5A). This analysis demonstrated a significant reduction in adhesion area for Fmn1-IV −/− MEFs, 1516±67.2 μm, compared to wild-type MEFs, 1865±98.7 μm (Fig. 5B). Since a cell plating defect could be accounted for by differences in focal adhesion formation, we next stained plated MEFs at different time intervals with antibody markers to focal adhesions. Immunofluorescence staining of MEFs with antibodies to phospho-tyrosine and phospho-caveolin, confirmed a reduction in focal adhesion formation at the leading edge of cells that lack Fmn1-IV (Fig. 6A). At 10 and 20 minutes post-plating, 39% and 31% of Fmn1-IV +/+ cells, and 7.4% and 7.8% of Fmn1-IV −/− cells formed greater than 15 focal adhesions per cell, respectively (Fig 6B). This points to a role for Fmn1-IV in the efficiency of focal adhesion formation, and may help explain the altered protrusive behavior of the Fmn1-IV null MEFs.

### Fmn1-IV does not significantly co-localize with adherens junctions of primary kidney epithelial cells

**Figure 7:**
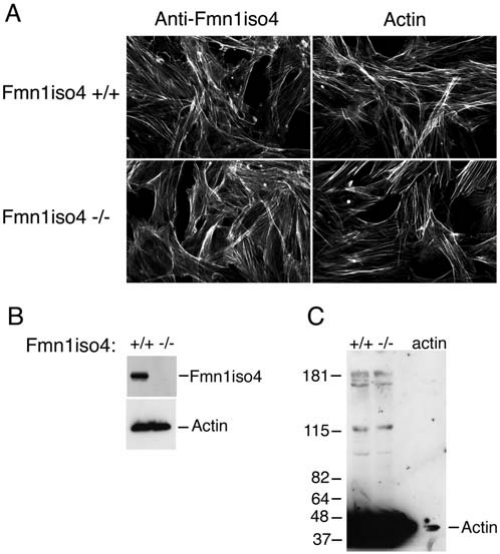
Rabbit polyclonal antibody raised against exon 6 of Fmn1-IV predominately reacts with actin. (A) MEFs derived from Fmn1-IV wild type and null mice were stained for immunofluorescence with anti-Fmn1-IV antibodies [29], [31] and phalloidin, as indicated at the top of the panels. Note that the staining patterns are similar for all images. (B) Western blot of MEF extracts stained with anti-Fmn1 antibody [32], and actin as a loading control. (C) Western blot of MEF extracts and actin (last lane) stained with anti-Fmn1-IV antibody [29], [31].

**Figure 8:**
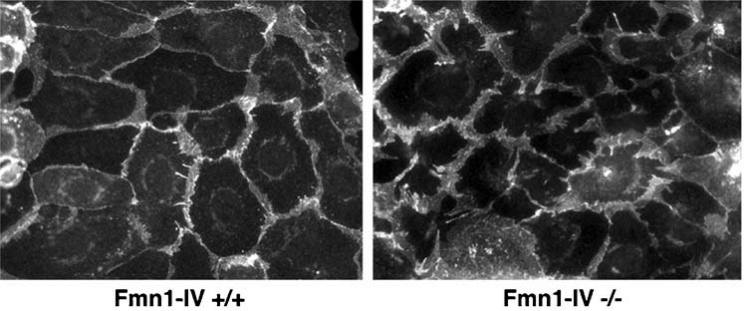
Formation of adherens junctions in Fmn1-IV null primary kidney epithelial cells. Primary kidney epithelial cells derived from Fmn1-IV wild type and null mice were stained for immunofluorescence with anti-E-cadherin antibodies.

A previous report had suggested that Fmn1-IV participates directly in adherens junctions in primary keratinocytes by use of dominant negative constructs for Fmn1-IV [29]. Treatment of MDCK cells with RNAi to Fmn1 also showed effects on cell-cell adhesions [30]. To re-examine this question, we looked to determine the localization of Fmn1-IV in primary kidney epithelial cells derived from EGFP-Fmn1-IV knock-in mice (Fig. 2) as described above. By co-staining epithelial cells with markers for adherens junction (E-cadherin and β-catenin) as well as actin, we were unable to observe any significant localization of Fmn1-IV to adherens junctions. This is in contrast to the complete overlapping localization of Fmn1-IV to adherens junctions observed in primary keratinocytes [29]. To understand the nature of this discrepancy, we requested the polyclonal antiserum produced against exon 6 of Fmn1-IV [31], which was used in the primary kerratinocyte study [29]. Immunofluorescence was then performed with the exon 6 antiserum on wild type and Fmn1-IV null MEFs. This revealed a similar staining pattern for both cells (Fig. 7A, left panels). This staining pattern was consistent with that of phalloidin, an actin stain (Fig. 7A, right panels). We were able to confirm that the cells used in our experiments were truly null for Fmn1-IV expression by performing a western blot on wild type and Fmn1-IV null MEFs with our Fmn1 antibody [32; Fig. 7B]. To address the issue of cross reactivity of the exon 6 antibody with actin, MEF extracts were again subjected to western blotting and purified β-actin was run side-by-side on a SDS-PAGE gel (Fig. 7C). The anti-serum directed against exon 6 of Fmn1 showed very strong reactivity to actin (43 kDa) with very little specific activity to Fmn1-IV (165 kDa). Although Fmn1-IV does not appear to localize significantly to adherens junctions, it remains possible that it may still have a regulatory role in adherens junction formation. In examining primary kidney epithelial cells, the Fmn1-IV null cells seemed to show a subtle delay in adherens junction formation as compared to wild type cells, but were ultimately able to form adherens junctions (Fig. 8). It is possible that Fmn1-IV could contribute to the efficiency of adherens junction formation through an indirect mechanism, such as proper initiation of cell-cell contact.

## Discussion

Given that there are a relatively large number of members of the formin family that may be expressed in any given cell type, their spatial control must be considered. In previous studies, it was shown that Fmn1-IV has a fairly broad tissue distribution pattern [25], [26], and that it is expressed both in fibroblasts and epithelial cells. In this study we show through the deletion of Fmn1-IV that it contributes to cell spreading and focal adhesion formation. The subcellular localization of Fmn1-IV within the cytoplasm and along microtubules suggests that it might be involved in the transport of factors that play a role in focal adhesion formation.

Recent evidence has pointed to a coordinative link between the actin and microtubule cytoskeletal networks to direct proper polarization of cells. EB1 and APC have been identified as linking proteins to mDia and actin which stabilize microtubules [33]. The yeast formin protein, for3, complexes with the plus end microtubule protein, tea1p [34]. These studies suggest a role for formins in regulating polarization of cells through an actin/microtubule linkage. Here we have shown that Fmn1-IV localizes along microtubules. Unlike Formin1-isoform1 which has been shown to associate with microtubules *in vitro* through its N-terminal exon 2 encoded peptide [28], Fmn1-IV does not possess this coding region and has a distinct localization. We speculate that the association of Fmn1-IV with microtubules might be transient.

Cell motility involves the interpretation of extracellular signals by cell surface receptors and subsequent downstream signals. One consequence is the well-characterized activation of the Rho family of GTPases which regulate numerous targets, some of which affect actin cytoskeleton organization [35]. Rho has been shown to act through mDia in the stabilization of microtubules [21], [36]. Microtubule stabilization at the leading edge is thought to proceed the extension of lamellipodia during cell migration, a process in which mDia1 is involved [21], [33]. Coordination of actin and microtubule filaments through the Rho-mDia1 pathway appear to deliver APC to the front of migrating cells for the induction of cell polarization [37]. FHOD1, another formin family member, acts through Rac and enhances cell migration when overexpressed [38]. Also, it has been shown that FRL enhances cell motility [39]. Although we demonstrated a role for Fmn1-IV in cell motility, it is not clear that the Rho family GTPases directly regulates it. Fmn1-IV does not possess a consensus GTPase binding domain, however, it remains possible that Fmn1-IV might be activated by Rho GTPases indirectly.

Close examination of Fmn1-IV localization shows it not to be directly at the leading edge of the cell or at the tips of filopodia, but rather within the cytoplasm. This may suggest that Fmn1-IV is not directly contributing to the processivity of the cell periphery, but rather is involved in forming or maintaining filamentous actin of a less prominent nature. The contribution of Fmn1-IV to efficient focal adhesion formation would be predicted to play a role in proper cell spreading. Studies examining macrophages derived from focal adhesion kinase (FAK) null mice, demonstrated elevated protrusion rates coupled with reduced cell spreading and cell adhesion dynamics [40]. The FAK−/− phenotype is similar to that observed in the current study on Fmn1-IV, however, in contrast Fmn1-IV does not appear to be significantly localizing to sites of focal adhesion. This is distinct from mDia1 in that it is believed to act directly on focal adhesion turnover to influence cell motility. Reduced cell spreading and focal adhesion formation in Fmn1-IV −/− MEFs further demonstrates the role of Formin family members in cellular processes that involve regulation of actin nucleation.

## Materials and Methods

### Cell lines and materials

MEFs, and primary kidney epithelial cells were maintained at 37°C in Dulbecco's Modified Eagle's Medium (Gibco-BRL), supplemented with 10% fetal calf serum (FBS) and penicillin-streptomycin. Anti-GFP rabbit polyclonal antibody (Abcam; and gift from Pam Silver, Dana-Farber Cancer Institute), anti-GFP mouse monoclonal antibody (Santa Cruz), anti-Actin mouse monoclonal antibody (Sigma), anti-α-Tubulin rabbit polyclonal antibody (Abcam), anti-E-cadherin antibody, anti-β-catenin antibody, anti-phospho-caveolin (BD Transduction Lab), and anti-phospho-tyrosine(4G10; Upstate). Generation of anti-Fmn1 rabbit polyclonal antibody has been described previously [32]. Rabbit polyclonal antibodies generated against exon 6 of Fmn1-IV were provided by Lloyd Cantley [31].

### Preparation of primary cells

MEFs were prepared by harvesting embryonic stage 14.5 mice. Placenta and membranes were removed from the embryo, and the visceral tissue was removed. The remaining embryonic tissue was placed in 0.25% trypsin-EDTA (BRL/Gibco) and miced with a sterile razor blade, followed by triturated with a glass pipet. Large cell aggregates were allowed to sediment for 5 min in a 15 ml conical tube, and the single cell suspended supernatant was then transferred to a new tube and pelleted by centrifugation. MEFs were then plated on fibronectin (Sigma) coated glass coverslips for immunofluorescence, or into 6-well dishes for later harvest of cell lysates for western blotting.

Primary kidney epithelial cells were prepared by harvesting kidneys from 6 day-old mice. Whole kidneys were placed in 0.25% trypsin-EDTA (BRL/Gibco) overnight, miced, triturated, and single cell suspension prepared as with MEFs. Kidney epithelial cells were then plated at appropriate densities on poly-L-lysine coated glass coverslips and allowed to plate for 2 days. Cells were then treated with 0.25% trypsin-EDTA to remove any fibroblasts attached to the coverslips, while leaving the epithelial cells attached. Primary epithelial cells were further incubated in preparation for immunofluorescence.

### Time-lapse microscopy and cell motility analysis

Sequential time-lapse images of MEFs were performed by allowing cells to grow to confluence on fibronectin coated glass chamber slides (Nalge Nunc). Monolayers were disrupted with a pipette tip and cells were allowed to migrate into the wound for 4 hrs. Images were taken at 10 sec. intervals for a total of 100 images under a 20× objective with a Zeiss Axiovert 200 M using Openlab (Improvision) software. Dynamics of the cell periphery were quantitated by kymographic analysis using ImageJ (National Institutes of Health) to determine time and space plots. Kymographs of active cell protrusion and retraction was performed by use of the segment line tool along the area of movement. The change in distance (μm, Δy) and persistence (min, Δx) was calculated through the length and angle of the line for each protrusion or retraction. The data were exported to Excel (Microsoft) for statistical analysis. Protrusion rates (Δy/Δx) and retraction rates (−Δy/Δx) were determined in μm/min. The data represent the mean±SEM of 53 (wild-type) and 57 (Fmn1-IV −/−) cell periphery events over 3 separate experiments.

### Immunofluorescence staining

MEFs and primary kidney epithelial cells were grown on fibronectin and poly-lysine -coated glass coverslips, respectively, and were fixed with 3.7% formaldehyde in PBS at 37°C for 5 min. Cells were permeabilized with 0.2% Saponin, 0.1% BSA, in PBS at RT throughout reagent staining. Primary antibodies were incubated on permeabilized cells for 1 h at RT, followed by incubation with Alexa Fluor conjugated secondary antibodies or conjugated phalloidin (Molecular Probes) for 30 min at RT. Stained cells were mounted with Fluoromount-G (Southern Biotechnology Associates, Inc.). Images were taken using a 40× oil immersion objective with a Zeiss Axiovert 200 M using Openlab (Improvision) software.

### Cell spreading and focal adhesion analysis

MEFs were propagated at low passage number in the presence of DMEM with 10% FBS. Both for cell spreading and focal adhesion analysis MEFs were re-plated at a density of 100,000 cells per 6-well dish on fibronectin-coated coverslips. For examination of cell spreading, MEFs were fixed after 15 min of plating in 3.7% formaldehyde and stained for filamentous actin with conjugated phalloidin. The adhesion area was determined using Openlab (Improvision) by the measurement of cell circumference. The data represent the mean±SEM of 100 (wild-type) and 100 (Fmn1-IV −/−) cells excluding cells with a circumference of <100 μm. MEFs were analysed for focal adhesion formation over varying time periods by immunofluorescence staining with an anti-phospho-tyrosine and anti-phospho-caveolin antibodies. Focal adhesions were counted per cell as phosphor-tyrosine positive staining at the leading edge of cells. Data was clustered in groups of 1–3, 4–9, 10–15, and >15 focal adhesions per cells and presented graphically in total percentages of cells with in each group.

### Statistical Analysis

Student's t-distribution was used assuming unequal variance to determine statistical significance.

### Strategy for insertion of the EGFP gene into the Formin 1 locus

Fusion targeting vector pNeo-EGFP-Fmn1-IV contains a Fmn1-IV genomic sequence from a BAC library. The 12 kb fragment covers the 5′UTR, exon 1 and exon 2 of Fmn1-IV. The EGFP gene (from pEGFP-C2 plasmid, Clontech) and PGKNeo cassette was inserted at the ATG start site (star) of exon1 of Formin1-IV. Two LoxP sites are flanking in both sides of PGK-Neo cassette. Mice were crossed with a cre expressing line to remove the PGK-Neo. Genotypes were confirmed by Southern blot and PCR analysis. PCR was performed using standard methods with primers flanking EGFP at the 5′ end and Fmn1-IV exon 6 at the 3′ end: 5′ CAAAGACCCCAACGAGAAGC and 5′ AGGGATTTCATAGAGAATTTAG. Expression of the EGFP-Fmn1-IV allele was confirmed by western blot and immunofluorescence.

### Electron Microscopy

GFP-Fmn1-IV knock-in MEFs and 129 MEFs (as wild type control) grown on plastic coverslips. Cells were permeabilized in a saponin solution (0.05 mg/ml) for 5 min at 37°C. After saponin treatment, cells were fixed in a mixture of 2% paraformaldehyde and 0.1% glutaraldehyde and 0.05 mg/ml of saponin in PHEM (60 mM Pipes, 25 mM Hepes, 10 mM EGTA and 2 mM MgCl2, pH 6.9) at 37°C for 20 min. They were rinsed with PHEM. Cells were then socked in 33% methanol in PHEM at RT for 10 min followed by a PHEM rinse. Blocking was performed with 0.5% fish skin gelatin in antibody dilution buffer (AbDil; 0.1 % Triton X-100 / 2% BSA) and incubated at RT for one hour. Then primary antibodies were applied, anti-EGFP polyclonal IgG (Pam Silver), anti-mouse tubulin IgG at 1:50 and 1:100 dilution in AbDil buffer at RT for 2 hrs. For detection of the primary antibodies, protein A coupled to 15 nm gold particles or anti mouse IgG labeled with 6 nm gold particles (Aurion) were used at dilutions 1:65 and 1: 20 at RT for 30 min. After the last incubation, cells were rinsed once in AbDil buffer, then five times in 0.05 M cacodylate buffer. Cells were fixed in 1.25% glutaraldehyde in 0.05 M cacodylate buffer at RT for 10 min. Followed by three times cacodylate buffer rinse. In dark, 1% osmium in 0.8% K_3_Fe(CN)_6_ were used for post-fixation, leave on ice for 15 min, then cells were washed, dehydrated, and processed for EM.
